# Exposure to polycyclic aromatic hydrocarbons, heavy metals, and per- and polyfluoroalkyl substances and their associations with serum lipid profiles in the general Korean adult population

**DOI:** 10.1186/s12940-025-01185-4

**Published:** 2025-05-11

**Authors:** Sanghee Shin, Youlim Kim, Yunsoo Choe, Su Hwan Kim, Jaelim Cho, Changsoo Kim, Kyoung-Nam Kim

**Affiliations:** 1 Department of Preventive Medicine, Yonsei University College of Medicine, 50-1 Yonsei-ro, Seodaemun-gu, Seoul, 03722 Republic of Korea; 2 Department of Pediatrics, Hanyang University College of Medicine, Seoul, Republic of Korea; 3 Department of Information Statistics, Gyeongsang National University, Jinju, Republic of Korea; 4 Institute for Environmental Research, Yonsei University College of Medicine, Seoul, Republic of Korea; 5 Institute of Human Complexity and Systems Science, Yonsei University, Incheon, Republic of Korea

**Keywords:** Polycyclic aromatic hydrocarbons, Heavy metals, Per- and polyfluoroalkyl substances, Lipid profile

## Abstract

**Background:**

Previous studies on associations between polycyclic aromatic hydrocarbons (PAHs) and lipid profiles are limited. We investigated the associations between urinary PAH metabolites and serum lipid profiles using a representative sample of Korean adults.

**Methods:**

This study utilized data from the Korean National Environmental Health Survey (2018–2020) (*n* = 2,516). The associations of PAH metabolites, heavy metals, and per- and polyfluoroalkyl substances (PFASs), which are ubiquitous pollutants, with lipid indicators and dyslipidemia types were evaluated using linear and logistic regression models, respectively. We examined the associations between a mixture of PAH metabolites, heavy metals, and PFASs and lipid profiles using quantile g-computation analyses.

**Results:**

A doubling of 1-hydroxypyrene (1-OHP) concentrations was associated with higher total cholesterol (TC) [β = 2.50 mg/dL, 95% confidence interval (CI): 1.09, 3.91], low-density lipoprotein cholesterol (LDL-C) (β = 2.39 mg/dL, 95% CI: 1.14, 3.63), and non-high-density lipoprotein cholesterol (non-HDL-C) concentrations (β = 2.13 mg/dL, 95% CI: 0.77, 3.49). A doubling of 1-OHP concentrations was also linked to higher odds of high TC [odds ratio (OR) = 1.15, 95% CI: 1.02, 1.30]. Additionally, 2-naphthol concentrations were associated with higher odds of high TC (OR = 1.14, 95% CI: 1.00, 1.29) and high LDL-C (OR = 1.27, 95% CI: 1.06, 1.51). Lead concentrations were associated with higher levels of TC, LDL-C, non-HDL-C, and high-density lipoprotein cholesterol (HDL-C), as well as with higher odds of high TC, high LDL-C, and high non-HDL-C. Mercury concentrations were associated with higher levels of TC, LDL-C, and non-HDL-C, and with higher odds of high TC. Several PFASs, such as perfluorooctanoic acid, perfluorononanoic acid, and perfluorodecanoic acid, were also associated with lipid profiles. A mixture of PAH metabolites, heavy metals, and PFASs was associated with higher TC, LDL-C, non-HDL-C, and HDL-C concentrations. This mixture was also linked to higher odds of high TC and high LDL-C.

**Conclusion:**

Concentrations of PAH metabolites, heavy metals, and PFASs were associated with unfavorable lipid profiles in the general adult population.

**Supplementary Information:**

The online version contains supplementary material available at 10.1186/s12940-025-01185-4.

## Background

Dyslipidemia, characterized by unfavorable serum lipid profiles, is a well-established risk factor for cardiovascular disease, one of the leading causes of mortality and morbidity worldwide [1]. The prevalence of dyslipidemia among adults is notably high, affecting 40–50% of the population in the United States [2], 79% in India [3], 34% in China [4], and 37.7–45.4% in the Republic of Korea [5].

In addition to traditional risk factors such as an unhealthy diet, physical inactivity, obesity, and tobacco smoking, environmental pollutants—such as polycyclic aromatic hydrocarbons (PAHs), heavy metals, and per- and polyfluoroalkyl substances (PFASs)—have been identified as emerging contributors to unfavorable lipid profiles. PAHs, a class of semi-volatile organic compounds, are predominantly generated through the incomplete combustion of organic matter. They are widespread in the environment and commonly originate from vehicle emissions and the burning of fossil fuels [6]. Heavy metals, such as lead, mercury, and cadmium, are recognized as major public health concerns due to their diverse sources of exposure and their ability to cause harmful health effects, even at low concentrations. PFASs are synthetic chemicals widely used in consumer products—such as non-stick cookware, food packaging, and water-repellant fabrics—and in industrial applications, owing to their exceptional resistance to water, oil, and heat [7].

The level of epidemiological evidence for the associations between these pollutants and serum lipid profiles differs by pollutant. Although previous studies have generally reported associations between heavy metals and PFASs and lipid profiles [8–11], evidence for PAHs is more limited; a few studies investigating the association have reported unfavorable lipid profiles associated with PAH exposure, but the results have been heterogeneous in terms of specific exposures and outcomes [12–14]. In addition, although people are simultaneously exposed to these ubiquitous chemicals in real-world settings, and shared underlying mechanisms (e.g., pollutant-induced oxidative stress and alterations in gut microbiome composition) [15–18] may enhance the effects of co-exposure, studies examining the effects of their combined exposure remain limited.

Therefore, we investigated the associations of PAH metabolites, heavy metals, and PFASs with serum lipid profiles using a representative sample of Korean adults. In addition, to reflect a more realistic exposure context, we explored the impact of co-exposure to PAHs, heavy metals, and PFASs on lipid profiles through a mixture analysis method.

## Methods

### Study population

This study utilized data from the Korean National Environmental Health Survey (KoNEHS), an ongoing cross-sectional survey conducted since 2009 by the Korean Ministry of Environment. The survey aims to gather comprehensive information on pollutant exposure levels, as well as the sociodemographic and behavioral factors influencing these levels within the Korean population. To obtain pollutant exposure values representative of the Korean population, the survey employs a two-stage, proportionally stratified sampling design. It involves conducting face-to-face interviews using structured questionnaires and collecting blood and urine samples from participants [19].

Among 4,239 adult participants from the latest available cycle of KoNEHS (the fourth cycle, 2018–2020), we excluded 1,245 individuals without lipid indicator data, 183 with triglyceride (TG) levels of 400 mg/dL or higher [to accurately estimate low-density lipoprotein cholesterol (LDL-C) concentrations using the Friedewald formula] [20], and 295 who reported using lipid-lowering medications. Consequently, the final sample consisted of 2,516 participants.

The KoNEHS survey was approved by the Institutional Review Board (IRB) of the National Institute of Environmental Research (Approval No. NIER-2018-BR-003-02), and all participants provided written informed consent. Additionally, the present study received ethical approval from the IRB of Severance Hospital (Approval No. 4-2024-0588).

### Measurement of pollutant levels

Spot urine and blood samples, collected without considering fasting status during the survey, were transferred to a deep freezer within 24 h at 2–6℃ and stored at -70℃. Frozen samples were transported to an analytical laboratory while maintaining their frozen state.

Detailed methods of analysis have been presented previously for PAH metabolites, heavy metals [21], and PFASs [22]. Briefly, urinary concentrations of PAH metabolites—1-hydroxypyrene (1-OHP), 2-naphthol (2-NAP), 1-hydroxyphenanthrene (1-OHPhe), and 2-hydroxyfluorene (2-OHFlu)—were determined after enzymatic hydrolysis using β-glucuronidase, followed by liquid-liquid extraction and analysis with a Clarus 680T gas chromatography-mass spectrometer (PerkinElmer, USA). For heavy metals, blood lead and urine cadmium concentrations were measured using an Analyst 800 graphite furnace atomic absorption spectrometer (PerkinElmer, USA) after appropriate dilution, while blood mercury concentrations were determined without pretreatment using a direct mercury analyzer with gold amalgamation (DMA-80, Milestone, Italy). Serum concentrations of five legacy PFASs—perfluorooctanoic acid (PFOA), perfluorooctane sulfonic acid (PFOS), perfluorohexane sulfonic acid (PFHxS), perfluorononanoic acid (PFNA), and perfluorodecanoic acid (PFDeA)—were quantified after solid-phase extraction followed by analysis with a QSight Triple Quad high-performance liquid chromatography/mass spectrometer (PerkinElmer, USA).

The limits of detection (LODs) for these pollutants and the proportion of samples with concentrations below their respective LODs are summarized in Table S1. Concentrations below the LOD were imputed as the LOD divided by √2.

### Lipid indicators and dyslipidemia

Serum total cholesterol (TC), high-density lipoprotein cholesterol (HDL-C), and TG levels were analyzed using an ADVIA 1800 Auto Analyzer (Siemens Healthineers, USA) with the enzymatic method, elimination/catalase method, and glycerol phosphate oxidase-Trinder method, respectively. Concentrations of TC, HDL-C, and TG were measured from serum samples collected without consideration of fasting status. Although TC and HDL-C levels have been reported to be unaffected by fasting status, TG levels may be affected [23]; therefore, the results for TG in this study should be interpreted with caution. LDL-C concentrations were determined using the Friedewald equation [24]. Because increased TG levels after dietary intake can reduce the accuracy of estimated LDL-C levels calculated using the Friedewald equation, we excluded participants with TG levels of 400 mg/dL or higher, as recommended [20]. Non-high-density lipoprotein cholesterol (non-HDL-C) concentrations—a screening marker that is reliable regardless of fasting status or elevated TG levels—were calculated by subtracting HDL-C (mg/dL) from TC (mg/dL).

For dyslipidemia, high TC was defined as TC ≥ 240 mg/dL, high LDL-C as LDL-C ≥ 160 mg/dL, high non-HDL-C as non-HDL-C ≥ 190 mg/dL, low HDL-C as HDL-C < 40 mg/dL for men and < 50 mg/dL for women, and high TG as TG ≥ 200 mg/dL [25].

### Covariates

Based on prior research on related topics [8–14], we identified potential confounders and predictors of blood lipids that are neither mediators nor colliders. These variables were adjusted for in all analyses: age (year), sex (male or female), educational level (≤ middle school, high school, or ≥ college or university), married or cohabiting (no or yes), tobacco smoking (never smoker, past smoker, or current smoker), alcohol consumption (no or yes), regular exercise (no, exercise but not to the point of sweating, or sweat-inducing exercise), and body mass index (kg/m^2^, < 18.5, 18.5–22.9, 23.0–24.9, or ≥ 25.0). Information on these variables, including height and weight, was collected using structured questionnaires. However, for 34 participants, height and weight were measured during surveys due to variations in survey procedures across different sites. No missing values were observed for all covariates.

### Statistical analysis

We accounted for the urine dilution effect in the exposure assessment of PAHs and cadmium by applying the covariate-adjusted standardization and covariate adjustment method [26]. Creatinine levels were predicted using a linear regression model that incorporated age, sex, and body mass index as predictors.

PAH metabolite, heavy metal, and PFAS concentrations were log-transformed using base 2 to approximate normal distributions, as they followed log-normal distributions, and the log-transformed values were used in subsequent analyses.

We performed a Pearson correlation analysis to evaluate the correlations among the log-transformed concentrations of PAH metabolites, heavy metals, and PFASs. We then evaluated the associations of individual PAH metabolites, heavy metals, and PFASs with continuous lipid indicators (TC, LDL-C, non-HDL-C, HDL-C, and TG levels) and binary dyslipidemia types (high TC, high LDL-C, high non-HDL-C, low HDL-C, and high TG) using linear and logistic regression models, respectively. The SURVEYREG and SURVEYLOGISTIC procedures in SAS software were employed, incorporating appropriate strata, cluster, and weight variables.

Because previous studies have reported more pronounced effects of pollutants on lipid profiles in older adults [8] and differences in effects by sex [27, 28], we conducted stratified analyses based on age (< 65 vs. ≥ 65 years) and sex. We chose 65 years as the cut-off for the age-stratified analysis because it is an established threshold for older adults according to the World Health Organization and many countries, including Korea [29].

We then evaluated the associations of a PAH metabolite, heavy metal, and PFAS mixture with lipid indicators and dyslipidemia types using quantile g-computation models with Gaussian and binomial distributions for the outcomes, respectively [30]. Pollutant concentrations were categorized into quartiles, and the associations, adjusted for the same set of covariates, were assessed using 300 bootstrap iterations. The results of the quantile g-computation analyses can be interpreted as covariate-adjusted association estimates for lipid indicators and dyslipidemia types, based on simultaneous increase in all pollutants by one quartile. We also evaluated the relative importance of each pollutant in terms of its impact on lipid profiles by calculating its weight contribution to the mixture effects.

As a sensitivity analysis, we repeated the linear regression analysis for TC, LDL-C, non-HDL-C, and HDL-C levels, and the logistic regression analysis for high TC, high LDL-C, high non-LDL-C, and low HDL-C, without excluding the 183 individuals with TG levels of 400 mg/dL or higher.

We followed the STROBE (Strengthening the Reporting of Observational Studies in Epidemiology) guidelines to ensure transparent and complete reporting of this observational study [31].

## Results

The mean age of the study participants was 51.3 years. Of the participants, 57.2% were female, 46.1% had a college or university education or higher, and 44.4% had a body mass index of 25.0 kg/m^2^ or higher. Compared to participants without any type of dyslipidemia (i.e., those without any of the following conditions: high TC, high LDL-C, high non-HDL-C, low HDL-C, or high TG, as defined based on the measured lipid indicators), those with any type of dyslipidemia (i.e., those with at least one of these abnormalities) had a higher mean age (53.5 years vs. 49.7 years), a lower educational level (40.7% vs. 50.1% with a college or university education or higher), and a higher body mass index (54.5% vs. 36.9% with a body mass index of 25.0 kg/m^2^ or higher) (Table 1).

**Table 1:** Characteristics of study participants

Variables	Total (*n* = 2,516)	Dyslipidemia (*n* = 1,078)	No dyslipidemia (*n* = 1,438)
Age (year)	51.3 ± 14.9	53.5 ± 13.9	49.7 ± 15.5
Sex			
Male	1,078 (42.9)	474 (44.0)	604 (42.0)
Female	1,438 (57.2)	604 (56.0)	834 (58.0)
Educational level			
≤ Middle school	628 (25.0)	311 (28.9)	317 (22.0)
High school	729 (29.0)	328 (30.4)	401 (27.9)
≥ College or university	1,159 (46.1)	439 (40.7)	720 (50.1)
Married or cohabiting			
No	606 (24.1)	243 (22.5)	363 (25.2)
Yes	1,910 (75.9)	835 (77.5)	1,075 (74.8)
Tobacco smoking			
Never smoker	1,649 (65.5)	686 (63.6)	963 (67.0)
Past smoker	483 (19.2)	210 (19.5)	273 (19.0)
Current smoker	384 (15.3)	182 (16.9)	202 (14.1)
Alcohol consumption			
No	532 (21.1)	268 (24.9)	264 (18.4)
Yes	1,984 (78.9)	810 (75.1)	1,174 (81.6)
Regular exercise			
No	1,356 (53.9)	581 (53.9)	775 (53.9)
Exercise but not to the point of sweating	170 (6.8)	91 (8.4)	79 (5.5)
Sweat-inducing exercise	990 (39.4)	406 (37.7)	584 (40.6)
Body mass index (kg/m^2^)			
< 18.5	51 (2.0)	11 (1.0)	40 (2.8)
18.5–22.9	741 (29.5)	209 (19.4)	532 (37.0)
23.0–24.9	607 (24.1)	271 (25.1)	336 (23.4)
≥ 25.0	1,117 (44.4)	587 (54.5)	530 (36.9)

The geometric means and distributions of PAH metabolite, heavy metal, and PFAS concentrations, which are generally comparable to or slightly higher than those observed in the United States [32], Canada [33–35], and Germany [36–38], are presented in Table S2. When we assessed the correlations among pollutants, heavy metals and PFASs were positively correlated with each other, showing varying strengths. However, PAHs were relatively independent of PFASs and heavy metals, with ρ ranging from − 0.12 to 0.11 (Table S3).

After adjusting for covariates, a doubling of 1-OHP concentrations was associated with a 2.50 mg/dL increase in TC [95% confidence interval (CI): 1.09, 3.91], 2.39 mg/dL increase in LDL-C (95% CI: 1.14, 3.63), and 2.13 mg/dL increase in non-HDL-C (95% CI: 0.77, 3.49). A doubling of lead concentrations was associated with increases of 7.22 mg/dL in TC (95% CI: 4.31, 10.12), 4.20 mg/dL in LDL-C (95% CI: 2.08, 6.33), 5.51 mg/dL in non-HDL-C (95% CI: 2.60, 8.42), and 1.70 mg/dL in HDL-C (95% CI: 0.56, 2.85). Mercury concentrations were associated with increases of 4.40 mg/dL in TC (95% CI: 1.72, 7.00), 3.46 mg/dL in LDL-C (95% CI: 1.17, 5.74), and 3.61 mg/dL in non-HDL-C (95% CI: 1.30, 5.92). Doubling PFOA concentrations was associated with a 3.70 mg/dL increase in LDL-C concentrations (95% CI: 1.01, 6.38). A doubling of PFNA concentrations was linked to increases of 4.64 mg/dL in TC (95% CI: 0.50, 8.79), 4.56 mg/dL in LDL-C (95% CI: 2.24, 6.88), and 3.96 mg/dL in non-HDL-C (95% CI: 0.75, 7.17). For PFDeA, a doubling was associated with a 4.92 mg/dL increase in LDL-C (95% CI: 1.72, 8.14) (Table 2).

**Table 2:** Associations of polycyclic aromatic hydrocarbons, heavy metals, and per- and polyfluoroalkyl substances with lipid indicators among community-dwelling adults

Pollutants	TC	LDL-C	Non-HDL-C	HDL-C	TG
	β (95% CI)	β (95% CI)	β (95% CI)	β (95% CI)	β (95% CI)
1-OHP	2.50 (1.09, 3.91)	2.39 (1.14, 3.63)	2.13 (0.77, 3.49)	0.37 (-0.09, 0.83)	-1.29 (-3.36, 0.78)
2-NAP	1.03 (-1.08, 3.14)	0.94 (-0.93, 2.81)	0.87 (-0.95, 2.68)	0.17 (-0.44, 0.78)	-0.36 (-3.52, 2.80)
1-OHPhe	0.53 (-0.62, 1.68)	0.60 (-0.83, 2.03)	0.13 (-1.04, 1.31)	0.40 (-0.11, 0.90)	-2.33 (-5.37, 0.70)
2-OHFlu	0.004 (-1.79, 1.80)	-0.30 (-2.00, 1.40)	-0.16 (-1.89, 1.57)	0.16 (-0.35, 0.68)	0.69 (-2.48, 3.87)
Lead	7.22 (4.31, 10.12)	4.20 (2.08, 6.33)	5.51 (2.60, 8.42)	1.70 (0.56, 2.85)	6.54 (-0.46, 13.53)
Mercury	4.40 (1.72, 7.00)	3.46 (1.17, 5.74)	3.61 (1.30, 5.92)	0.75 (-0.21, 1.72)	0.76 (-5.11, 6.63)
Cadmium	-0.62 (-3.06, 1.82)	0.26 (-1.48, 2.00)	-0.51 (-2.55, 1.54)	-0.11 (-1.13, 0.91)	-3.83 (-10.06, 2.41)
PFOA	3.82 (-1.83, 9.47)	3.70 (1.01, 6.38)	2.95 (-1.48, 7.39)	0.87 (-0.70, 2.43)	-3.72 (-16.73, 9.28)
PFOS	2.00 (-3.26, 7.26)	1.82 (-1.65, 5.29)	2.43 (-1.81, 6.69)	-0.44 (-1.92, 1.05)	3.10 (-3.28, 9.47)
PFHxS	1.13 (-1.50, 3.76)	1.29 (-0.74, 3.32)	0.60 (-1.69, 2.83)	0.56 (-0.23, 1.35)	-3.61 (-11.63, 4.41)
PFNA	4.64 (0.50, 8.79)	4.56 (2.24, 6.88)	3.96 (0.75, 7.17)	0.68 (-0.78, 2.15)	-3.00 (-13.38, 7.38)
PFDeA	4.81 (-0.74, 10.35)	4.92 (1.72, 8.14)	3.63 (-0.53, 7.79)	1.18 (-0.93, 3.28)	-6.49 (-15.83, 2.84)

Abbreviations: TC, total cholesterol; LDL-C, low-density lipoprotein cholesterol; Non-HDL-C, non-high-density lipoprotein cholesterol; HDL-C, high-density lipoprotein cholesterol; TG, triglyceride; CI, confidence interval; 1-OHP, 1-hydroxypyrene; 2-NAP, 2-naphthol; 1-OHPhe, 1-hydroxyphenanthrene; 2-OHFlu, 2-hydroxyfluorene; PFOA, perfluorooctanoic acid; PFOS, perfluorooctane sulfonic acid; PFHxS, perfluorohexane sulfonic acid; PFNA, perfluorononanoic acid; PFDeA, perfluorodecanoic acid. Regression coefficients (β) and 95% confidence intervals were estimated using linear regression models incorporating appropriate strata, cluster, and weight variables. The models were adjusted for age, sex, educational level, married or cohabiting, tobacco smoking, alcohol consumption, regular exercise, and body mass index

In the analyses of dyslipidemia types as outcomes, a doubling of 1-OHP concentrations was associated with higher odds of high TC [odds ratio (OR) = 1.15, 95% CI: 1.02, 1.30], and a doubling of 2-NAP concentrations was linked to higher odds of high TC (OR = 1.14, 95% CI: 1.00, 1.29) and high LDL-C (OR = 1.27, 95% CI: 1.06, 1.51). However, a doubling of 1-OHPhe concentrations was associated with lower odds of low HDL-C (OR = 0.91, 95% CI: 0.83, 0.99). A doubling of lead concentrations was linked to higher odds of high TC (OR = 1.54, 95% CI: 1.25, 1.88), high LDL-C (OR = 1.44, 95% CI: 1.02, 2.04), and high non-HDL-C (OR = 1.48, 95% CI: 1.04, 2.12). Mercury concentrations showed an association with higher odds of high TC (OR = 1.26, 95% CI: 1.003, 1.59). A doubling of PFOA concentrations (OR = 1.50, 95% CI: 1.05, 2.15), PFNA concentrations (OR = 1.45, 95% CI: 1.07, 1.96), and PFDeA concentrations (OR = 1.53, 95% CI: 1.02, 2.30) was associated with higher odds of high TC (Table 3).

**Table 3:** Associations of per- and polyfluoroalkyl substances, heavy metals, and polycyclic aromatic hydrocarbons with dyslipidemia types among community-dwelling adults

Pollutants	High TC	High LDL-C	High non-HDL-C	Low HDL-C	High TG
	OR (95% CI)	OR (95% CI)	OR (95% CI)	OR (95% CI)	OR (95% CI)
1-OHP	1.15 (1.02, 1.30)	1.12 (0.91, 1.37)	1.14 (0.96, 1.34)	0.97 (0.90, 1.04)	0.97 (0.90, 1.04)
2-NAP	1.14 (1.00, 1.29)	1.27 (1.06, 1.51)	1.14 (0.98, 1.33)	0.98 (0.87, 1.10)	0.96 (0.88, 1.05)
1-OHPhe	0.98 (0.88, 1.09)	0.97 (0.84, 1.12)	0.91 (0.77, 1.07)	0.91 (0.83, 0.99)	0.96 (0.89, 1.04)
2-OHFlu	0.90 (0.74, 1.07)	0.83 (0.61, 1.12)	0.79 (0.62, 1.01)	1.06 (0.95, 1.20)	1.04 (0.95, 1.14)
Lead	1.54 (1.25, 1.88)	1.44 (1.02, 2.04)	1.48 (1.04, 2.12)	0.82 (0.65, 1.03)	1.08 (0.83, 1.40)
Mercury	1.26 (1.003, 1.59)	1.43 (0.99, 2.05)	1.24 (0.89, 1.72)	0.92 (0.78, 1.10)	0.98 (0.82, 1.15)
Cadmium	0.98 (0.79, 1.21)	0.99 (0.77, 1.27)	0.97 (0.74, 1.27)	1.17 (0.97, 1.41)	0.87 (0.72, 1.05)
PFOA	1.50 (1.05, 2.15)	1.36 (0.93, 1.98)	1.31 (0.96, 1.80)	0.86 (0.72, 1.04)	0.89 (0.62, 1.27)
PFOS	1.22 (0.88, 1.69)	1.04 (0.79, 1.37)	1.30 (0.84, 2.02)	1.08 (0.88, 1.34)	1.06 (0.85, 1.31)
PFHxS	1.01 (0.80, 1.27)	0.93 (0.72, 1.21)	0.93 (0.75, 1.15)	0.90 (0.81, 1.01)	0.87 (0.71, 1.06)
PFNA	1.45 (1.07, 1.96)	1.25 (0.87, 1.79)	1.39 (0.95, 2.04)	0.89 (0.72, 1.10)	0.88 (0.66, 1.19)
PFDeA	1.53 (1.02, 2.30)	1.36 (0.84, 2.20)	1.48 (0.92, 2.38)	0.88 (0.66, 1.18)	0.77 (0.59, 1.02)

Abbreviations: TC, total cholesterol; LDL-C, low-density lipoprotein cholesterol; Non-HDL-C, non-high-density lipoprotein cholesterol; HDL-C, high-density lipoprotein cholesterol; TG, triglyceride; CI, confidence interval; 1-OHP, 1-hydroxypyrene; 2-NAP, 2-naphthol; 1-OHPhe, 1-hydroxyphenanthrene; 2-OHFlu, 2-hydroxyfluorene; PFOA, perfluorooctanoic acid; PFOS, perfluorooctane sulfonic acid; PFHxS, perfluorohexane sulfonic acid; PFNA, perfluorononanoic acid; PFDeA, perfluorodecanoic acid. Odds ratios and 95% confidence intervals were estimated using logistic regression models incorporating appropriate strata, cluster, and weight variables. The models were adjusted for age, sex, educational level, married or cohabiting, tobacco smoking, alcohol consumption, regular exercise, and body mass index

The age- and sex-stratified analyses revealed no evidence of heterogeneity in the associations of PAH metabolites, heavy metals, and PFASs with lipid profiles by age and sex, as indicated by overlapping CIs. However, the point estimates of the associations were generally higher among individuals aged ≥ 65 years than those aged < 65 years, particularly for continuous lipid indicators (Tables S4 and S5).

In the quantile g-computation analyses, simultaneous increases in PAH metabolite, heavy metal, and PFAS concentrations by one quartile were associated with increases of 8.03 mg/dL in TC concentrations (95% CI: 4.69, 11.36; driven by PFOA with a weight of 0. 30 and mercury with a weight of 0.20), 6.33 mg/dL in LDL-C concentrations (95% CI: 3.43, 9.23; driven by PFOA with a weight of 0.24 and mercury with a weight of 0.19), 6.47 mg/dL in non-HDL-C concentrations (95% CI: 3.39, 9.56; driven by PFOA with a weight of 0.26 and mercury with a weight of 0.21), and 1.56 mg/dL in HDL-C concentrations (95% CI: 0.28, 2.83; driven by PFDeA with a weight of 0.44 and PFOA with a weight of 0.17). Increasing pollutant mixture concentrations by one quartile was also associated with higher odds of high TC (OR = 1.63, 95% CI: 1.20, 2.21; driven by PFDeA with a weight of 0.34 and lead with a weight of 0.20) and high LDL-C (OR = 1.69, 95% CI: 1.12, 2.53; driven by PFDeA with a weight of 0.46 and cadmium with a weight of 0.18) (Tables S6 and S7).

Sensitivity analyses including individuals with TG levels of 400 mg/dL or higher yielded robust results that were consistent with those of the main analyses (Tables S8 and S9).

## Discussion

In the general adult population, 1-OHP concentrations were associated with higher TC, LDL-C, and non-HDL-C concentrations, as well as higher odds of high TC. 2-NAP concentrations were associated with higher odds of high TC and high LDL-C. Meanwhile, 1-OHPhe concentrations were associated with lower odds of low HDL-C. A mixture of PAH metabolites, heavy metals, and PFASs—ubiquitous pollutants worldwide—was linked to higher TC, LDL-C, non-HDL-C, and HDL-C concentrations. This mixture was also associated with higher odds of high TC and high LDL-C. The present study addresses research gaps related to the limited evidence on the association between PAH metabolites and lipid profiles, as well as on the association between co-exposure to PAH metabolites, heavy metals, and PFASs and lipid profiles, thereby reflecting a more realistic exposure context.

Previous studies on the associations between PAHs and lipid profiles are relatively rare and have provided heterogeneous results. For example, 2-NAP, 1-OHPhe, and 2-OHFlu, but not 1-OHP, concentrations were reported to be associated with dyslipidemia in studies using National Health and Nutrition Examination Survey (NHANES) data [13, 39]. Meanwhile, a study conducted among the Chinese general population reported that 1-OHPhe and 2-OHFlu concentrations were associated with higher odds of high TC and high LDL-C, respectively, while 1-OHP and 2-NAP were not associated with any type of dyslipidemia [40]. Given the limited existing research and the inconsistent findings across studies, further investigation is needed to conclusively characterize the associations between PAHs and lipid profiles.

A previous study using NHANES data reported that blood lead, but not cadmium, concentrations were associated with higher odds of high TC, high LDL-C, and high non-HDL-C [41], consistent with the present study’s results. The associations between lead and unfavorable lipid profiles were consistently demonstrated in other studies [10, 11]. However, although the present study and the previous study using NHANES data [41] did not find the associations between cadmium and lipid profiles, these associations were reported in a few studies [9, 10], possibly due to correlations between cadmium and lead and/or residual confounding. Additionally, our findings regarding the associations between mercury and lipid profiles align with those of previous studies examining the relationships [9, 27].

Despite heterogeneity in the associations between specific PFAS substances and lipid indicators reported in some studies [42, 43], a recent systematic review demonstrated sufficient evidence linking PFOA, PFOS, and PFNA to higher TC concentrations, as well as PFOS and perfluoroundecanoic acid to higher LDL-C concentrations [8]. These findings partially align with the results of the present study, which identified associations between PFOA and PFNA and high TC.

There have been a limited number of studies investigating the health impacts of chemical mixtures consisting of various classes of pollutants (e.g., PAH, heavy metal, and PFAS). For example, co-exposure to PAHs and PFASs was reported to be associated with higher total bilirubin, TC, and LDL-C concentrations in the population of professional firefighters and controls [44]. PAH and heavy metal mixtures were demonstrated to be associated with thyroid hormone levels [21] and depression [45]. However, although liver and thyroid function (and related depression) are closely related to lipid metabolism [46], to the best of our knowledge, no previous study has examined the joint associations of PAH metabolites, heavy metals, and PFASs with lipid profiles.

In addition to the specific pathways for each substance, shared biological mechanisms through which the considered pollutants (PAHs, heavy metals, and PFASs) influence serum lipid levels can be proposed. Pollutant-induced oxidative stress may lead to lipid peroxidation, hepatocellular ferroptosis, disruption of lipid metabolism, and unfavorable lipid profiles [15, 16]. Moreover, many pollutants can alter the composition of the gut microbiota, which is closely linked to lipid metabolism, thereby affecting lipid profiles [17, 18].

The following limitations should be considered to properly interpret the results of this study. First, the cross-sectional design of this study precludes establishing causal relationships. Second, some biomarkers of the considered pollutants, measured in a single spot urine or blood sample (e.g., PAH metabolites), may not accurately reflect long-term exposure levels [47]. Third, although factors such as increased health awareness, healthy lifestyles, and high dietary quality may act as confounding variables, they were only indirectly accounted for through variables such as educational level, tobacco smoking, alcohol consumption, regular exercise, and body mass index. Therefore, concerns about residual confounding remain. Fourth, factors that may affect lipid profiles, such as a family history of dyslipidemia and underlying diseases, could not be considered due to data limitations. Fifth, because we conducted multiple analyses, some observed associations may have occurred by chance due to an inflated α error, and the results should be interpreted with caution.

However, this study also offers several advantages that merit acknowledgment. First, by examining three classes of ubiquitously present pollutants, our approach provides more realistic exposure scenarios and their health effects in the community. Second, because we used carefully designed data to represent the Korean adult population, concerns about selection bias were minimized. Third, we utilized a wide range of meticulously collected variables, all of which were of high quality and free of missing values.

## Conclusions

Concentrations of PAH metabolites, heavy metals, and PFASs were associated with unfavorable lipid profiles in the general adult population. Additionally, a mixture of PAH metabolites, heavy metals, and PFASs was associated with unfavorable lipid profiles. While this study analyzed three classes of commonly encountered pollutants, reflecting realistic exposure scenarios and their associated health effects, its cross-sectional design limits the ability to establish causality. Future longitudinal studies are needed to confirm these findings and strengthen the evidence for causal relationships.

## Electronic supplementary material

Below is the link to the electronic supplementary material.

Supplementary Material 1

## Data Availability

No datasets were generated or analysed during the current study.
