# Supplementary material for: Exposure to polycyclic aromatic hydrocarbons, heavy metals, and per- and polyfluoroalkyl substances and their associations with serum lipid profiles in the general Korean adult population
Source: Environ Health. 2025 May 11;24:30. doi: 10.1186/s12940-025-01185-4 (PMC12067673; doi:10.1186/s12940-025-01185-4)
Supplement: Supplementary file 1 — Supplementary Material 1 [file 12940_2025_1185_MOESM1_ESM.docx]

*Supplementary Material*

**Exposure to polycyclic aromatic hydrocarbons, heavy metals, and per- and polyfluoroalkyl substances and their associations with serum lipid profiles in the general Korean adult population**

Sanghee Shin, Youlim Kim, Yunsoo Choe, Su Hwan Kim, Jaelim Cho, Changsoo Kim, Kyoung-Nam Kim

**Table of Contents**

**Table S1.** The limits of detection for the analyzed pollutants and the proportion of samples with concentrations below these limits

**Table S2.** The geometric means and distributions of polycyclic aromatic hydrocarbon metabolite, heavy metal, and per- and polyfluoroalkyl substance concentrations

**Table S3.** Correlations among chemical concentrations in a Pearson correlation analysis

**Table S4.** Associations of polycyclic aromatic hydrocarbon metabolites, heavy metals, and per- and polyfluoroalkyl substances with lipid indicators, stratified by age (< 65 vs. ≥ 65 years) and sex

**Table S5.** Associations of polycyclic aromatic hydrocarbon metabolites, heavy metals, and per- and polyfluoroalkyl substances with dyslipidemia types, stratified by age (< 65 vs. ≥ 65 years) and sex

**Table S6.** Associations of a polycyclic aromatic hydrocarbon, heavy metal, and per- and polyfluoroalkyl substance mixture with lipid indicators and dyslipidemia types in quantile g-computation analyses

**Table S7.** Positive and negative weight contributions of pollutants in the quantile g-computation analysis

**Table S8.** Associations of polycyclic aromatic hydrocarbons, heavy metals, and per- and polyfluoroalkyl substances with lipid indicators among community-dwelling adults, including individuals with triglyceride levels of 400 mg/dL or higher

**Table S9.** Associations of per- and polyfluoroalkyl substances, heavy metals, and polycyclic aromatic hydrocarbons with dyslipidemia types among community-dwelling adults, including individuals with triglyceride levels of 400 mg/dL or higher

**Table S1.** The limits of detection for the analyzed pollutants and the proportion of samples with concentrations below these limits

|  | LOD | Below the LOD^a^ |
| --- | --- | --- |
| 1-OHP | 0.015 μg/L | 14 (0. 6%) |
| 2-NAP | 0.050 μg/L | 1 (0.04%) |
| 1-OHPhe | 0.040 μg/L | 611 (24.3%) |
| 2-OHFlu | 0.040 μg/L | 74 (2.9%) |
| Lead | 0.170 μg/dL | 5 (0.2%) |
| Mercury | 0.100 μg/L | 5 (0.2%) |
| Cadmium | 0.040 μg/L | 37 (1.5%) |
| PFOA | 0.050 μg/L | 0 (0%) |
| PFOS | 0.056 μg/L | 0 (0%) |
| PFHxS | 0.071 μg/L | 2 (0.1%) |
| PFNA | 0.019 μg/L | 1 (0.04%) |
| PFDeA | 0.017 μg/L | 0 (0%) |

Abbreviations: LOD, limit of detection; 1-OHP, 1-hydroxypyrene; 2-NAP, 2-naphthol; 1-OHPhe, 1-hydroxyphenanthrene; 2-OHFlu, 2-hydroxyfluorene; PFOA, perfluorooctanoic acid; PFOS, perfluorooctane sulfonic acid; PFHxS, perfluorohexane sulfonic acid; PFNA, perfluorononanoic acid; PFDeA, perfluorodecanoic acid.

^a^Values are presented as *n* (%).

**Table S2.** The geometric means and distributions of polycyclic aromatic hydrocarbon metabolite, heavy metal, and per- and polyfluoroalkyl substance concentrations

| Pollutants | GM | GSD | Percentile | | | | |
| --- | --- | --- | --- | --- | --- | --- | --- |
|  |  |  | 5th | 25th | 50th | 75th | 95th |
| 1-OHP (μg/L) | 0.2 | 3.2 | 0.02 | 0.1 | 0.3 | 0.5 | 1.1 |
| 2-NAP (μg/L) | 3.3 | 2.7 | 0.8 | 1.6 | 2.8 | 6.6 | 19.7 |
| 1-OHPhe (μg/L) | 0.1 | 3.3 | 0.02 | 0.04 | 0.1 | 0.3 | 0.7 |
| 2-OHFlu (μg/L) | 0.3 | 2.6 | 0.6 | 0.2 | 0.3 | 0.6 | 1.9 |
| Lead (μg/dL) | 1.6 | 1.6 | 0.7 | 1.2 | 1.6 | 2.1 | 3.3 |
| Mercury (μg/L) | 3.1 | 2.0 | 1.1 | 1.9 | 3.0 | 4.7 | 9.6 |
| Cadmium (μg/L) | 0.6 | 2.2 | 0.1 | 0.4 | 0.7 | 1.0 | 1.7 |
| PFOA (μg/L) | 6.9 | 1.8 | 2.8 | 4.7 | 7.0 | 10.0 | 16.9 |
| PFOS (μg/L) | 16.3 | 1.8 | 6.0 | 10.7 | 16.6 | 24.6 | 42.8 |
| PFHxS (μg/L) | 4.3 | 2.1 | 1.4 | 2.7 | 4.2 | 6.7 | 14.9 |
| PFNA (μg/L) | 2.3 | 1.9 | 0.8 | 1.5 | 2.4 | 3.6 | 6.2 |
| PFDeA (μg/L) | 1.0 | 1.8 | 0.4 | 1.0 | 1.0 | 1.5 | 2.5 |

Abbreviations: GM, geometric mean; GSD, geometric standard deviation; 1-OHP, 1-hydroxypyrene; 2-NAP, 2-naphthol; 1-OHPhe, 1-hydroxyphenanthrene; 2-OHFlu, 2-hydroxyfluorene; PFOA, perfluorooctanoic acid; PFOS, perfluorooctane sulfonic acid; PFHxS, perfluorohexane sulfonic acid; PFNA, perfluorononanoic acid; PFDeA, perfluorodecanoic acid.

**Table S3.** Correlations among chemical concentrations in a Pearson correlation analysis

|  | 2-NAP | 1-OHPhe | 2-OHFlu | Lead | Mercury | Cadmium | PFOA | PFOS | PFHxS | PFNA | PFDeA |
| --- | --- | --- | --- | --- | --- | --- | --- | --- | --- | --- | --- |
| 1-OHP | 0.31  (<.0001) | 0.37  (<.0001) | 0.34  (<.0001) | 0.03  (0.10) | -0.03  (0.20) | 0.04  (0.06) | -0.04  (0.04) | -0.12  (<.0001) | -0.04  (0.03) | -0.05  (0.02) | -0.04  (0.04) |
| 2-NAP |  | 0.24  (<.0001) | 0.47  (<.0001) | 0.08  (<.0001) | 0.04  (<.05) | 0.02  (0.24) | -0.04  (0.03) | -0.10  (<.0001) | 0.03  (0.17) | -0.05  (0.01) | -0.06  (0.002) |
| 1-OHPhe |  |  | 0.34  (<.0001) | 0.07  (0.001) | 0.04  (0.07) | 0.08  (0.0001) | 0.03  (0.13) | -0.03  (0.50) | 0.01  (0.51) | 0.02  (0.22) | 0.01  (0.60) |
| 2-OHFlu |  |  |  | 0.11  (<.0001) | 0.01  (0.62) | 0.05  (0.02) | -0.01  (0.50) | -0.05  (0.02) | 0.07  (0.001) | -0.01  (0.54) | -0.03  (0.19) |
| Lead |  |  |  |  | 0.25  (<.0001) | 0.19  (<.0001) | 0.32  (<.0001) | 0.31  (<.0001) | 0.25  (<.0001) | 0.38  (<.0001) | 0.36  (<.0001) |
| Mercury |  |  |  |  |  | 0.12  (<.0001) | 0.40  (<.0001) | 0.40  (<.0001) | 0.24  (<.0001) | 0.51  (<.0001) | 0.53  (<.0001) |
| Cadmium |  |  |  |  |  |  | 0.26  (<.0001) | 0.25  (<.0001) | 0.13  (<.0001) | 0.33  (<.0001) | 0.30  (<.0001) |
| PFOA |  |  |  |  |  |  |  | 0.67  (<.0001) | 0.55  (<.0001) | 0.83  (<.0001) | 0.72  (<.0001) |
| PFOS |  |  |  |  |  |  |  |  | 0.42  (<.0001) | 0.80  (<.0001) | 0.78  (<.0001) |
| PFHxS |  |  |  |  |  |  |  |  |  | 0.46  (<.0001) | 0.43  (<.0001) |
| PFNA |  |  |  |  |  |  |  |  |  |  | 0.92  (<.0001) |

Abbreviations: PFOA, perfluorooctanoic acid; PFOS, perfluorooctane sulfonic acid; PFHxS, perfluorohexane sulfonic acid; PFNA, perfluorononanoic acid; PFDeA, perfluorodecanoic acid; 1-OHP, 1-hydroxypyrene; 2-NAP, 2-naphthol; 1-OHPhe, 1-hydroxyphenanthrene; 2-OHFlu, 2-hydroxyfluorene.

All pollutant concentrations were log_2_-transformed. Values are presented as the correlation coefficient ρ (*p*-value).

**Table S4.** Associations of polycyclic aromatic hydrocarbon metabolites, heavy metals, and per- and polyfluoroalkyl substances with lipid indicators, stratified by age (< 65 vs. ≥ 65 years) and sex

|  | TC | LDL-C | Non-HDL-C | HDL-C | TG |
| --- | --- | --- | --- | --- | --- |
|  | β (95% CI) | β (95% CI) | β (95% CI) | β (95% CI) | β (95% CI) |
| < 65 years (*n* = 1,970) | | | | | |
| 1-OHP | 1.91 (0.63, 3.19) | 1.72 (0.61, 2.82) | 1.72 (0.49, 2.95) | 0.19 (-0.31, 0.70) | 0.01(-2.51, 2.52) |
| 2-NAP | 0.86 (-1.04, 2.77) | 0.71 (-0.84, 2.25) | 0.76 (-0.86, 2.39) | 0.10 (-0.56, 0.75) | 0.27 (-3.29, 3.83) |
| 1-OHPhe | 0.64 (-0.51,1.79) | 0.39 (-0.92, 1.69) | 0.29 (-0.89, 1.46) | 0.35 (-0.14, 0.85) | -0.52 (-3.40, 2.36) |
| 2-OHFlu | -0.43 (-2.44, 1.58) | -0.84 (-2.77, 1.10) | -0.44 (-2.38, 1.50) | 0.01 (-0.58, 0.50) | 1.97 (-1.64, 5.57) |
| Lead | 4.94 (2.07, 7.81) | 2.17 (0.08, 4.26) | 3.36 (0.40, 6.31) | 1.58 (0.26, 2.90) | 5.93 (-2.49, 14.34) |
| Mercury | 1.72 (-1.71, 5.16) | 1.34 (-1.16, 3.85) | 1.23 (-1.67, 4.12) | 0.50 (-0.70, 1.70) | -0.57 (-7.18, 6.03) |
| Cadmium | -1.58 (-3.99, 0.83) | -0.67 (-2.47, 1.14) | -1.54 (-3.69, 0.62) | -0.04 (-1.05, 0.97) | -4.34 (-10.99, 2.30) |
| PFOA | 1.62 (-4.75, 8.00) | 1.80 (-2.23, 5.83) | 0.73 (-4.45, 5.90) | 0.90 (-0.80, 2.60) | -5.37 (-15.31, 4.56) |
| PFOS | 0.63 (-4.31, 5.57) | 0.95 (-2.41, 4.32) | 1.15 (-3.16, 5.47) | -0.52 (-2.00, 0.95) | 1.01 (-7.09, 9.11) |
| PFHxS | -0.88 (-4.42, 2.65) | -0.57 (-3.27, 2.13) | -1.23 (-4.31, 1.85) | 0.34 (-0.67, 1.36) | -3.29 (-9.08, 2.50) |
| PFNA | 2.39 (-2.22, 7.00) | 2.84 (-0.07, 5.77) | 1.89 (-1.69, 5.48) | 0.49 (-1.16, 2.15) | -4.72 (-13.43, 3.98) |
| PFDeA | 2.82 (-3.30, 8.94) | 3.84 (-0.04, 7.72) | 1.61 (-3.08, 6.30) | 1.21 (-1.07, 3.49) | -11.13 (-20.47, -1.79) |
| ≥ 65 years (*n* = 546) | | | | | |
| 1-OHP | 3.07 (-0.22, 6.37) | 2.38 (-0.59, 5.35) | 2.21 (-1.05, 5.47) | 0.86 (0.07, 1.66) | -0.84 (-5.61, 3.93) |
| 2-NAP | 2.45 (-2.33, 7.24) | 2.25 (-2.67, 7.17) | 2.12 (-2.14, 6.39) | 0.33 (-1.02, 1.68) | -0.63 (-7.23, 5.97) |
| 1-OHPhe | 0.45 (-2.35, 3.26) | 0.54 (-2.54, 3.62) | -0.02 (-2.63, 2.58) | 0.48 (-0.29, 1.25) | -2.80 (-6.62, 1.02) |
| 2-OHFlu | 2.35 (-3.60, 8.30) | 1.45 (-3.19, 6.10) | 1.42 (-3.76, 6.60) | 0.93 (-0.20, 2.07) | -0.17 (-6.86, 6.53) |
| Lead | 9.21 (-0.17, 18.59) | 7.49 (-0.62, 15.60) | 7.95 (-0.31, 16.21) | 1.26 (-1.18, 3.70) | 2.28 (-12.68, 17.25) |
| Mercury | 6.82 (1.43, 12.20) | 5.72 (1.13, 10.31) | 5.20 (0.75, 9.65) | 1.62 (0.001, 3.23) | -2.59 (-9.14, 3.95) |
| Cadmium | -0.53 (-4.87, 3.82) | 1.89 (-2.87, 6.64) | 0.38 (-3.78, 4.55) | -0.91(-2.53, 0.71) | -7.51 (-16.14, 1.12) |
| PFOA | 4.67 (0.08, 9.26) | 5.40 (1.43, 9.36) | 3.59 (-0.74, 7.92) | 1.08 (-0.05, 2.20) | -9.04 (-32.84, 14.77) |
| PFOS | 2.80 (-4.74, 10.33) | 1.57 (-4.12, 7.27) | 2.65 (-2.49, 7.79) | 0.15 (-2.96, 3.26) | 5.38 (-3.66, 14.41) |
| PFHxS | 3.89 (0.55, 7.23) | 4.22 (1.02, 7.42) | 2.22 (-0.88, 5.33) | 1.67 (0.82, 2.52) | -9.97 (-27.71, 7.77) |
| PFNA | 4.52 (-1.39, 10.42) | 5.13 (0.20, 10.06) | 2.74 (-2.21, 7.70) | 1.77 (-0.18, 3.73) | -11.92 (-33.55, 9.70) |
| PFDeA | 3.39 (-2.33, 9.10) | 2.81 (-1.47, 7.08) | 2.33 (-2.66, 7.33) | 1.05 (-0.70, 2.81) | -2.37 (-15.56, 10.83) |
| Male (*n* = 793) | | | | | |
| 1-OHP | 3.36 (1.41, 5.31) | 2.82 (0.98, 4.66) | 2.98 (1.10, 4.87) | 0.38 (-0.40, 1.15) | 0.83 (-3.66, 5.31) |
| 2-NAP | -0.29 (-2.79, 2.22) | 0.37 (-1.78, 2.53) | -0.56 (-2.99, 1.87) | 0.27 (-0.55, 1.08) | -4.65 (-10.83, 1.53) |
| 1-OHPhe | 1.81 (-0.03. 3.65) | 1.22 (-0.56, 3.01) | 1.06 (-0.83, 2.96) | 0.75 (0.13, 1.38) | -0.81 (-5.54, 3.93) |
| 2-OHFlu | 0.25 (-3.24, 3.74) | -0.09 (-3.17, 3.00) | 0.18 (-3.07, 3.43) | 0.07 (-0.77, 0.91) | 1.33 (-3.66, 6.33) |
| Lead | 7.28 (2.01, 12.56) | 3.18 (-1.07, 7.42) | 4.60 (-0.58, 9.77) | 2.69 (1.05, 4.32) | 7.10 (-4.72, 18.93) |
| Mercury | -0.80 (-6.93, 5.34) | -0.73 (-5.40, 3.95) | -0.65 (-5.92, 4.62) | -0.14 (-1.96, 1.67) | 0.37 (-11.20, 11.95) |
| Cadmium | -1.28 (-4.82, 2.25) | -0.90 (-3.87, 2.08) | -1.56 (-4.54, 1.41) | 0.28 (-1.24, 1.80) | -3.32 (-11.43, 4.78) |
| PFOA | -0.37 (-10.86, 10.12) | -0.08 (-6.61, 6.45) | -1.58 (-9.60, 6.44) | 1.21 (-1.88, 4.31) | -7.52 (-22.71, 7.66) |
| PFOS | -0.55 (-8.02, 6.91) | 0.24 (-4.91, 5.39) | 0.19 (-6.93, 7.31) | -0.75 (-2.72, 1.22) | -0.25 (-15.40, 14.89) |
| PFHxS | -2.28 (-7.61, 3.05) | -1.34 (-5.63, 2.97) | -2.37 (-7.01, 2.27) | 0.09 (-1.38, 1.56) | -5.17 (-12.87, 2.54) |
| PFNA | 0.24 (-7.99, 8.47) | 0.62 (-4.73, 5.97) | -0.15 (-6.85, 6.54) | 0.39 (-2.14, 2.93) | -3.87 (-19.31, 11.58) |
| PFDeA | 2.18 (-6.72, 11.08) | 3.36 (-2.23, 8.95) | 1.70 (-5.48, 8.88) | 0.48 (-2.64, 3.60) | -8.27 (-24.37, 7.83) |
| Female (*n* = 1,723) | | | | | |
| 1-OHP | 1.82 (-0.19, 3.83) | 1.60 (-0.001, 3.19) | 1.52 (-0.31, 3.36) | 0.29 (-0.31, 0.90) | -0.36 (-3.05, 2.24) |
| 2-NAP | 2.00 (-0.53, 4.53) | 1.42 (-0.99, 3.84) | 1.88 (-0.31, 4.07) | 0.12 (-0.61, 0.84) | 2.30 (-1.06, 5.65) |
| 1-OHPhe | -0.31 (-1.77, 1.15) | -0.19 (-1.91, 1.53) | -0.39 (-1.78, 1.00) | 0.08 (-0.52, 0.68) | -1.00 (-4.07, 2.07) |
| 2-OHFlu | 0.13 (-2.66, 2.92) | -0.31 (-2.82, 2.20) | 0.14 (-2.49, 2.77) | -0.02 (-0.67, 0.64) | 2.27 (-0.90, 5.45) |
| Lead | 6.43 (3.28, 9.57) | 4.22 (1.86, 6.59) | 5.33 (2.28, 8.38) | 1.10 (-0.19, 2.39) | 5.52 (-3.27, 14.32) |
| Mercury | 6.45 (3.22, 9.68) | 5.14 (2.37, 7.92) | 4.88 (2.16, 7.60) | 1.57 (0.41, 2.73) | -1.33 (-6.35, 3.69) |
| Cadmium | 0.50 (-1.83, 2.82) | 1.55 (-0.34, 3.45) | 0.79 (-1.41, 3.00) | -0.30 (-1.35, 0.75) | -3.80 (-10.31, 2.71) |
| PFOA | 5.91 (3.25, 8.56) | 5.59 (3.37, 7.81) | 4.89 (2.26, 7.53) | 1.01 (-0.04, 2.07) | -3.48 (-17.12, 10.16) |
| PFOS | 2.73 (-0.78, 6.23) | 2.07 (-0.74, 4.88) | 2.93 (0.25, 5.61) | -0.20 (-1.73, 1.33) | 4.28 (-1.66, 10.22) |
| PFHxS | 2.59 (0.29, 4.89) | 2.30 (-0.44, 5.03) | 1.59 (-0.49, 3.67) | 1.00 (0.13, 1.87) | -3.53 (-13.68, 6.63) |
| PFNA | 6.78 (4.12, 9.44) | 6.61 (3.50, 9.73) | 5.60 (3.04, 8.16) | 1.18 (0.05, 2.31) | -5.07 (-14.30, 4.16) |
| PFDeA | 6.15 (2.67, 9.63) | 5.89 (2.71, 9.07) | 4.42 (1.10, 7.75) | 1.73 (0.38, 3.07) | -7.34 (-14.11, -0.58) |

Abbreviations: TC, total cholesterol; LDL-C, low-density lipoprotein cholesterol; Non-HDL-C, non-high-density lipoprotein cholesterol; HDL-C, high-density lipoprotein cholesterol; TG, triglyceride; CI, confidence interval; 1-OHP, 1-hydroxypyrene; 2-NAP, 2-naphthol; 1-OHPhe, 1-hydroxyphenanthrene; 2-OHFlu, 2-hydroxyfluorene; PFOA, perfluorooctanoic acid; PFOS, perfluorooctane sulfonic acid; PFHxS, perfluorohexane sulfonic acid; PFNA, perfluorononanoic acid; PFDeA, perfluorodecanoic acid.

Regression coefficients (β) and 95% confidence intervals were estimated using linear regression models incorporating appropriate strata, cluster, and weight variables. The models were adjusted for age, sex, educational level, married or cohabiting, tobacco smoking, alcohol consumption, regular exercise, and body mass index.

**Table S5.** Associations of polycyclic aromatic hydrocarbon metabolites, heavy metals, and per- and polyfluoroalkyl substances with dyslipidemia types, stratified by age (< 65 vs. ≥ 65 years) and sex

|  | High TC | High LDL-C | High non-HDL-C | Low HDL-C | High TG |
| --- | --- | --- | --- | --- | --- |
|  | OR (95% CI) | OR (95% CI) | OR (95% CI) | OR (95% CI) | OR (95% CI) |
| < 65 years (*n* = 1,970) | | | | | |
| 1-OHP | 1.10 (0.96, 1.27) | 1.05 (0.84, 1.31) | 1.12 (0.94, 1.33) | 0.99 (0.92, 1.07) | 0.99 (0.93, 1.07) |
| 2-NAP | 1.15 (1.01, 1.31) | 1.25 (1.07, 1.47) | 1.14 (0.99, 1.32) | 1.03 (0.93, 1.15) | 0.98 (0.89, 1.08) |
| 1-OHPhe | 0.97 (0.88, 1.07) | 0.91 (0.78, 1.05) | 0.90 (0.78, 1.04) | 0.94 (0.86, 1.02) | 0.99 (0.92, 1.07) |
| 2-OHFlu | 0.87 (0.71, 1.07) | 0.75 (0.54, 1.05) | 0.77 (0.60, 0.98) | 1.15 (1.01, 1.31) | 1.05 (0.95, 1.18) |
| Lead | 1.36 (1.04, 1.78) | 1.31 (0.75, 2.30) | 1.35 (0.84, 2.19) | 0.85 (0.63, 1.15) | 1.02 (0.75, 1.38) |
| Mercury | 1.15 (0.87, 1.51) | 1.36 (0.90, 2.05) | 1.16 (0.78, 1.74) | 0.95 (0.75, 1.21) | 0.92 (0.74, 1.14) |
| Cadmium | 0.93 (0.75, 1.15) | 0.92 (0.74, 1.15) | 0.91 (0.70, 1.18) | 1.20 (0.96, 1.49) | 0.85 (0.69, 1.04) |
| PFOA | 1.37 (0.91, 2.08) | 1.28 (0.79, 2.08) | 1.19 (0.81, 1.77) | 0.85 (0.67, 1.07) | 0.82 (0.60, 1.14) |
| PFOS | 1.13 (0.77, 1.66) | 0.95 (0.66, 1.37) | 1.25 (0.74, 2.09) | 1.06 (0.82, 1.37) | 0.99 (0.77, 1.28) |
| PFHxS | 0.92 (0.69, 1.22) | 0.84 (0.58, 1.20) | 0.81 (0.60, 1.10) | 0.96 (0.83, 1.10) | 0.85 (0.68, 1.05) |
| PFNA | 1.38 (1.01, 1.88) | 1.21 (0.81, 1.82) | 1.35 (0.90, 2.05) | 0.92 (0.69, 1.24) | 0.80 (0.60, 1.07) |
| PFDeA | 1.41 (0.92, 2.15) | 1.31 (0.77, 2.23) | 1.43 (0.84, 2.43) | 0.85 (0.59, 1.23) | 0.66 (0.49, 0.90) |
| ≥ 65 years (*n* = 546) | | | | | |
| 1-OHP | 1.16 (0.94, 1.43) | 1.04 (0.75, 1.44) | 0.90 (0.66, 1.22) | 0.98 (0.85, 1.13) | 0.93 (0.80, 1.08) |
| 2-NAP | 0.93 (0.71, 1.22) | 1.40 (0.98, 2.00) | 0.90 (0.65, 1.26) | 0.85 (0.68, 1.08) | 0.97 (0.79, 1.20) |
| 1-OHPhe | 0.96 (0.81, 1.14) | 1.09 (0.91, 1.31) | 0.85 (0.69, 1.04) | 0.94 (0.82, 1.07) | 0.94 (0.83, 1.07) |
| 2-OHFlu | 0.79 (0.61, 1.04) | 0.96 (0.56, 1.65) | 0.74 (0.51, 1.09) | 0.87 (0.71, 1.07) | 1.05 (0.87, 1.27) |
| Lead | 2.51 (1.46, 4.29) | 1.64 (0.73, 3.70) | 1.54 (0.76, 3.14) | 0.74 (0.49, 1.12) | 1.11 (0.72, 1.72) |
| Mercury | 1.83 (1.28, 2.62) | 2.17 (1.47, 3.19) | 1.51 (1.07, 2.12) | 0.85 (0.66, 1.10) | 0.86 (0.68, 1.08) |
| Cadmium | 0.97 (0.63, 1.5) | 1.77 (0.95, 3.31) | 1.30 (0.74, 2.27) | 1.21 (0.92, 1.59) | 0.75 (0.57, 0.98) |
| PFOA | 1.81 (1.03, 3.19) | 2.08 (0.86, 5.04) | 1.67 (0.93, 3.03) | 0.90 (0.70, 1.15) | 0.76 (0.61, 0.97) |
| PFOS | 1.53 (0.97, 2.42) | 1.47 (0.81, 2.69) | 1.3 (0.81, 2.09) | 1.16 (0.72, 1.87) | 1.07 (0.78, 1.47) |
| PFHxS | 1.39 (0.94, 2.05) | 1.19 (0.84, 1.68) | 1.26 (0.95, 1.67) | 0.81 (0.68, 0.97) | 0.77 (0.65, 0.91) |
| PFNA | 1.02 (0.44, 2.39) | 1.38 (0.71, 2.66) | 0.97 (0.52, 1.78) | 0.78 (0.52, 1.16) | 0.72 (0.55, 0.95) |
| PFDeA | 1.92 (0.97, 3.81) | 1.55 (0.83, 2.90) | 1.22 (0.74, 2.02) | 0.97 (0.68, 1.37) | 0.82 (0.60, 1.12) |
| Male (*n* = 793) | | | | | |
| 1-OHP | 1.30 (1.05, 1.60) | 1.14 (0.80, 1.63) | 1.24 (1.01, 1.52) | 1.01 (0.84, 1.21) | 1.04 (0.94, 1.16) |
| 2-NAP | 0.96 (0.71, 1.31) | 1.13 (0.75, 1.71) | 0.98 (0.74, 1.30) | 1.00 (0.78, 1.27) | 0.87 (0.75, 1.00) |
| 1-OHPhe | 1.00 (0.83, 1.21) | 1.00 (0.80, 1.24) | 0.93 (0.75, 1.17) | 0.90 (0.79, 1.01) | 0.97 (0.87, 1.08) |
| 2-OHFlu | 0.85 (0.60, 1.21) | 0.92 (0.52, 1.61) | 0.79 (0.54, 1.17) | 1.17 (0.95, 1.44) | 1.07 (0.94, 1.22) |
| Lead | 1.21 (0.69, 2.13) | 1.05 (0.51, 2.18) | 1.02 (0.52, 2.01) | 0.96 (0.66, 1.40) | 0.91 (0.66, 1.25) |
| Mercury | 1.09 (0.69, 1.74) | 1.08 (0.65, 1.82) | 1.08 (0.64, 1.81) | 1.06 (0.80, 1.39) | 0.93 (0.74, 1.17) |
| Cadmium | 1.04 (0.80, 1.35) | 0.98 (0.72, 1.33) | 0.99 (0.73, 1.34) | 1.13 (0.88, 1.46) | 0.89 (0.72, 1.10) |
| PFOA | 1.51 (0.76, 3.02) | 1.16 (0.57, 2.39) | 1.26 (0.70, 2.25) | 0.74 (0.60, 0.91) | 0.80 (0.53, 1.21) |
| PFOS | 1.50 (0.94, 2.39) | 0.87 (0.55, 1.38) | 1.36 (0.78, 2.38) | 1.16 (0.83, 1.63) | 1.05 (0.82, 1.35) |
| PFHxS | 0.86 (0.62, 1.19) | 0.76 (0.5, 1.18) | 0.87 (0.61, 1.23) | 0.86 (0.71, 1.03) | 0.76 (0.62, 0.95) |
| PFNA | 1.34 (0.74, 2.43) | 0.81 (0.43, 1.51) | 1.15 (0.61, 2.18) | 0.92 (0.67, 1.26) | 0.86 (0.59, 1.24) |
| PFDeA | 1.46 (0.75, 2.88) | 0.86 (0.46, 1.61) | 1.22 (0.62, 2.41) | 0.92 (0.63, 1.36) | 0.81 (0.58, 1.13) |
| Female (*n* = 1,723) | | | | | |
| 1-OHP | 1.06 (0.91, 1.23) | 1.03 (0.78, 1.37) | 1.05 (0.80, 1.37) | 0.98 (0.89, 1.08) | 0.95 (0.86, 1.04) |
| 2-NAP | 1.26 (1.08, 1.45) | 1.36 (1.10, 1.69) | 1.28 (1.02, 1.59) | 0.97 (0.86, 1.09) | 1.06 (0.96, 1.18) |
| 1-OHPhe | 0.94 (0.85, 1.04) | 0.87 (0.7, 1.09) | 0.86 (0.70, 1.05) | 0.97 (0.89, 1.06) | 0.99 (0.89, 1.11) |
| 2-OHFlu | 0.95 (0.79, 1.13) | 0.72 (0.52, 1.00) | 0.81 (0.59, 1.11) | 1.03 (0.92, 1.15) | 1.04 (0.92, 1.18) |
| Lead | 1.69 (1.34, 2.13) | 1.77 (1.09, 2.87) | 1.95 (1.31, 2.92) | 0.73 (0.57, 0.92) | 1.28 (0.90, 1.83) |
| Mercury | 1.35 (1.10, 1.67) | 1.77 (1.17, 2.67) | 1.39 (0.95, 2.04) | 0.80 (0.65, 0.98) | 1.01 (0.85, 1.20) |
| Cadmium | 0.98 (0.78, 1.22) | 1.07 (0.85, 1.35) | 1.06 (0.83, 1.36) | 1.25 (0.99, 1.58) | 0.87 (0.70, 1.09) |
| PFOA | 1.45 (1.05, 1.99) | 1.55 (0.92, 2.61) | 1.33 (0.79, 2.21) | 1.00 (0.7, 1.42) | 1.01 (0.76, 1.34) |
| PFOS | 0.95 (0.71, 1.26) | 1.32 (0.80, 2.18) | 1.21 (0.73, 2.02) | 1.01 (0.75, 1.37) | 1.11 (0.84, 1.46) |
| PFHxS | 1.13 (0.92, 1.39) | 1.11 (0.84, 1.48) | 0.97 (0.71, 1.33) | 0.97 (0.81, 1.15) | 1.04 (0.84, 1.28) |
| PFNA | 1.46 (1.11, 1.92) | 1.89 (1.18, 3.04) | 1.66 (1.05, 2.62) | 0.86 (0.64, 1.14) | 0.92 (0.73, 1.16) |
| PFDeA | 1.64 (1.19, 2.26) | 2.33 (1.19, 4.56) | 1.99 (1.17, 3.37) | 0.83 (0.60, 1.14) | 0.73 (0.56, 0.94) |

Abbreviations: TC, total cholesterol; LDL-C, low-density lipoprotein cholesterol; Non-HDL-C, non-high-density lipoprotein cholesterol; HDL-C, high-density lipoprotein cholesterol; TG, triglyceride; OR, odds ratio; CI, confidence interval; 1-OHP, 1-hydroxypyrene; 2-NAP, 2-naphthol; 1-OHPhe, 1-hydroxyphenanthrene; 2-OHFlu, 2-hydroxyfluorene; PFOA, perfluorooctanoic acid; PFOS, perfluorooctane sulfonic acid; PFHxS, perfluorohexane sulfonic acid; PFNA, perfluorononanoic acid; PFDeA, perfluorodecanoic acid.

Odds ratios and 95% confidence intervals were estimated using logistic regression models incorporating appropriate strata, cluster, and weight variables. The models were adjusted for age, sex, educational level, married or cohabiting, tobacco smoking, alcohol consumption, regular exercise, and body mass index.

**Table S6.** Associations of a polycyclic aromatic hydrocarbon, heavy metal, and per- and polyfluoroalkyl substance mixture with lipid indicators and dyslipidemia types in quantile g-computation analyses

| Outcome | Regression coefficient^a^ | 95% confidence interval |
| --- | --- | --- |
| Lipid indicator |  |  |
| TC | 8.03 | 4.69, 11.36 |
| LDL-C | 6.33 | 3.43, 9.23 |
| Non-HDL-C | 6.47 | 3.39, 9.56 |
| HDL-C | 1.56 | 0.28, 2.83 |
| TG | 0.71 | -6.34, 7.76 |
|  |  |  |
| Dyslipidemia type |  |  |
| High TC | 1.63 | 1.20, 2.21 |
| High LDL-C | 1.69 | 1.12, 2.53 |
| High non-HDL-C | 1.26 | 0.84, 1.90 |
| Low HDL-C | 0.92 | 0.77, 1.11 |
| High TG | 0.99 | 0.85, 1.17 |

Abbreviations: TC, total cholesterol; LDL-C, low-density lipoprotein cholesterol; Non-HDL-C, non-high-density lipoprotein cholesterol; HDL-C, high-density lipoprotein cholesterol; TG, triglyceride.

^a^Results are presented as β estimates for lipid indicators and as odds ratios for dyslipidemia types.

The results were estimated using quantile g-computation models adjusted for age, sex, educational level, married or cohabiting, tobacco smoking, alcohol consumption, regular exercise, and body mass index.

**Table S7.** Positive and negative weight contributions of pollutants in the quantile g-computation analysis

| Pollutants | TC | LDL-C | Non-HDL-C | HDL-C | High TC | High LDL-C |
| --- | --- | --- | --- | --- | --- | --- |
| 1-OHP | 0.10 | 0.15 | 0.11 | -0.01 | 0.06 | -0.02 |
| 2-NAP | -0.13 | -0.13 | -0.18 | 0.04 | 0.03 | 0.13 |
| 1-OHPhe | -0.22 | -0.24 | -0.35 | 0.11 | -0.12 | 0.04 |
| 2-OHFlu | 0.02 | -0.03 | 0.004 | 0.03 | -0.12 | -0.23 |
| Lead | 0.20 | 0.10 | 0.18 | 0.10 | 0.20 | 0.03 |
| Mercury | 0.20 | 0.19 | 0.21 | 0.04 | -0.08 | 0.02 |
| Cadmium | -0.03 | 0.09 | 0.07 | -0.27 | 0.08 | 0.18 |
| PFOA | 0.30 | 0.24 | 0.26 | 0.17 | 0.19 | -0.04 |
| PFOS | 0.09 | 0.07 | 0.16 | -0.20 | 0.03 | 0.14 |
| PFHxS | 0.02 | 0.01 | -0.01 | 0.06 | 0.07 | -0.01 |
| PFNA | -0.61 | -0.60 | -0.17 | -0.51 | -0.68 | -0.70 |
| PFDeA | 0.08 | 0.15 | -0.29 | 0.44 | 0.34 | 0.46 |

Abbreviations: TC, total cholesterol; LDL-C, low-density lipoprotein cholesterol; Non-HDL-C, non-high-density lipoprotein cholesterol; HDL-C, high-density lipoprotein cholesterol; 1-OHP, 1-hydroxypyrene; 2-NAP, 2-naphthol; 1-OHPhe, 1-hydroxyphenanthrene; 2-OHFlu, 2-hydroxyfluorene; PFOA, perfluorooctanoic acid; PFOS, perfluorooctane sulfonic acid; PFHxS, perfluorohexane sulfonic acid; PFNA, perfluorononanoic acid; PFDeA, perfluorodecanoic acid.

The results were estimated using quantile g-computation models adjusted for age, sex, educational level, married or cohabiting, tobacco smoking, alcohol consumption, regular exercise, and body mass index.

**Table S8.** Associations of polycyclic aromatic hydrocarbons, heavy metals, and per- and polyfluoroalkyl substances with lipid indicators among community-dwelling adults, including individuals with triglyceride levels of 400 mg/dL or higher

| Pollutants | TC | LDL-C | Non-HDL-C | HDL-C |
| --- | --- | --- | --- | --- |
|  | β (95% CI) | β (95% CI) | β (95% CI) | β (95% CI) |
| 1-OHP | 2.20 (0.88, 3.73) | 2.79 (1.35, 4.24) | 1.91 (0.56, 3.27) | 0.39 (-0.06, 0.84) |
| 2-NAP | 1.15 (-1.00, 3.30) | 0.71 (-0.96, 2.37) | 1.04 (-0.87, 2.95) | 0.11 (-0.51, 0.73) |
| 1-OHPhe | 0.54 (-0.54, 1.63) | 0.62 (-1.11, 2.35) | 0.13 (-0.94, 1.19) | 0.42 (-0.03, 0.87) |
| 2-OHFlu | 0.23 (-1.48, 1.93) | -0.77 (-2.55, 1.00) | 0.15 (-1.52, 1.81) | 0.08 (-0.40, 0.57) |
| Lead | 7.09 (4.08, 10.10) | 2.92 (0.67, 5.17) | 5.41 (2.31, 8.52) | 1.68 (0.64, 2.71) |
| Mercury | 4.59 (2.14, 7.05) | 2.99 (0.10, 5.89) | 3.83 (1.70, 5.96) | 0.76 (-0.17, 1.69) |
| Cadmium | -0.09 (-2.55, 2.36) | -1.14 (-3.49, 1.21) | 0.07 (-2.00, 2.13) | -0.16 (-1.05, 0.73) |
| PFOA | 3.45 (-2.06, 8.95) | 4.30 (1.88, 6.73) | 2.34 (-1.97, 6.65) | 1.11 (-0.43, 2.64) |
| PFOS | 1.84 (-3.20, 6.89) | 2.58 (-0.94, 6.10) | 2.00 (-1.87, 5.88) | -0.16 (-1.76, 1.44) |
| PFHxS | 1.17 (-1.27, 3.62) | 1.02 (-0.99, 3.03) | 0.71 (-1.40, 2.82) | 0.46 (-0.31, 1.24) |
| PFNA | 4.39 (0.25, 8.53) | 5.23 (2.44, 8.01) | 3.36 (0.18, 6.54) | 1.03 (-0.37, 2.42) |
| PFDeA | 4.33 (-1.28, 9.93) | 5.71 (2.62, 8.80) | 2.76 (-1.46, 6.98) | 1.56 (-0.40, 3.53) |

Abbreviations: TC, total cholesterol; LDL-C, low-density lipoprotein cholesterol; Non-HDL-C, non-high-density lipoprotein cholesterol; HDL-C, high-density lipoprotein cholesterol; CI, confidence interval; 1-OHP, 1-hydroxypyrene; 2-NAP, 2-naphthol; 1-OHPhe, 1-hydroxyphenanthrene; 2-OHFlu, 2-hydroxyfluorene; PFOA, perfluorooctanoic acid; PFOS, perfluorooctane sulfonic acid; PFHxS, perfluorohexane sulfonic acid; PFNA, perfluorononanoic acid; PFDeA, perfluorodecanoic acid.

Regression coefficients (β) and 95% confidence intervals were estimated using linear regression models incorporating appropriate strata, cluster, and weight variables. The models were adjusted for age, sex, educational level, married or cohabiting, tobacco smoking, alcohol consumption, regular exercise, and body mass index.

**Table S9.** Associations of per- and polyfluoroalkyl substances, heavy metals, and polycyclic aromatic hydrocarbons with dyslipidemia types among community-dwelling adults, including individuals with triglyceride levels of 400 mg/dL or higher

| Pollutants | High TC | High LDL-C | High non-HDL-C | Low HDL-C |
| --- | --- | --- | --- | --- |
|  | OR (95% CI) | OR (95% CI) | OR (95% CI) | OR (95% CI) |
| 1-OHP | 1.12 (1.00, 1.27) | 1.12 (0.90, 1.39) | 1.08 (0.93, 1.26) | 0.95 (0.89, 1.02) |
| 2-NAP | 1.11 (0.99, 1.24) | 1.25 (1.05, 1.49) | 1.12 (0.99, 1.28) | 1.02 (0.92, 1.12) |
| 1-OHPhe | 0.99 (0.90, 1.08) | 0.97 (0.84, 1.12) | 0.93 (0.82, 1.05) | 0.91 (0.84, 0.99) |
| 2-OHFlu | 0.93 (0.80, 1.08) | 0.82 (0.60, 1.11) | 0.88 (0.73, 1.06) | 1.09 (0.98, 1.21) |
| Lead | 1.52 (1.26, 1.82) | 1.43 (1.01, 2.03) | 1.37 (0.96, 1.95) | 0.82 (0.66, 1.02) |
| Mercury | 1.28 (1.06, 1.55) | 1.40 (0.98, 2.00) | 1.28 (1.00, 1.65) | 0.91 (0.79, 1.07) |
| Cadmium | 1.02 (0.83, 1.27) | 0.96 (0.74, 1.25) | 1.02 (0.79, 1.30) | 1.18 (1.02, 1.37) |
| PFOA | 1.41 (1.04, 1.92) | 1.37 (0.95, 2.00) | 1.23 (0.95, 1.59) | 0.81 (0.67, 0.98) |
| PFOS | 1.15 (0.88, 1.50) | 1.05 (0.81, 1.38) | 1.16 (0.83, 1.63) | 1.02 (0.82, 1.28) |
| PFHxS | 1.01 (0.84, 1.21) | 0.92 (0.71, 1.19) | 0.97 (0.82, 1.15) | 0.92 (0.83, 1.03) |
| PFNA | 1.39 (1.09, 1.76) | 1.26 (0.90, 1.78) | 1.31 (0.99, 1.74) | 0.82 (0.67, 1.00) |
| PFDeA | 1.44 (1.04, 1.99) | 1.38 (0.88, 2.16) | 1.34 (0.94, 1.92) | 0.81 (0.62, 1.06) |

Abbreviations: TC, total cholesterol; LDL-C, low-density lipoprotein cholesterol; Non-HDL-C, non-high-density lipoprotein cholesterol; HDL-C, high-density lipoprotein cholesterol; CI, confidence interval; 1-OHP, 1-hydroxypyrene; 2-NAP, 2-naphthol; 1-OHPhe, 1-hydroxyphenanthrene; 2-OHFlu, 2-hydroxyfluorene; PFOA, perfluorooctanoic acid; PFOS, perfluorooctane sulfonic acid; PFHxS, perfluorohexane sulfonic acid; PFNA, perfluorononanoic acid; PFDeA, perfluorodecanoic acid.

Odds ratios and 95% confidence intervals were estimated using logistic regression models incorporating appropriate strata, cluster, and weight variables. The models were adjusted for age, sex, educational level, married or cohabiting, tobacco smoking, alcohol consumption, regular exercise, and body mass index.
